# A Randomized Controlled Trial of the Efficacy and Safety of CCX282-B, an Orally-Administered Blocker of Chemokine Receptor CCR9, for Patients with Crohn’s Disease

**DOI:** 10.1371/journal.pone.0060094

**Published:** 2013-03-20

**Authors:** Satish Keshav, Tomáš Vaňásek, Yaron Niv, Robert Petryka, Stephanie Howaldt, Mauro Bafutto, István Rácz, David Hetzel, Ole Haagen Nielsen, Séverine Vermeire, Walter Reinisch, Per Karlén, Stefan Schreiber, Thomas J. Schall, Pirow Bekker, the Prospective Randomized Oral-Therapy Evaluation in Crohn’s Disease Trial-1 (PROTECT-1) Study Group

**Affiliations:** 1 John Radcliffe Hospital, University of Oxford, Oxford, United Kingdom; 2 Hepato-Gastroenterologie HK, Hradec Králové, Czech Republic; 3 Rabin Medical Center, Petach Tikva, Israel; 4 NZOZ Vivamed, Zespół Lekarzy Specjalistów, Warszawa, Poland; 5 Praxis für Innere Medizin, Hamburg, Germany; 6 Instituto Goiano de Gastroenterologia e Endoscopia Digestiva Ltda., Goiânia, Brazil; 7 Petz Aladár County and Teaching Hospital, Győr, Hungary; 8 Royal Adelaide Hospital, Adelaide, Australia; 9 Department of Gastroenterology, Herlev Hospital, Herlev, Denmark; 10 UZ Gasthuisberg, Leuven, Belgium; 11 Allgemeines Krankenhaus Wien, Universitätsklinik für Innere Medizin III, Klinische Abteilung für Gastroenterologie und Hepatologie, Vienna, Austria; 12 Department of Clinical Science and Education, Karolinska Institutet, Södersjukhuset, Stockholm, Sweden; 13 Department of Medicine I, Christian Albrechts University, University Hospital Schleswig Holstein, Kiel, Germany; 14 ChemoCentryx, Inc., Mountain View, California, United States of America; Copenhagen University Hospital Gentofte, Denmark

## Abstract

CCX282-B, also called vercirnon, is a specific, orally-administered chemokine receptor CCR9 antagonist that regulates migration and activation of inflammatory cells in the intestine. This randomized, placebo-controlled trial was conducted to evaluate the safety and efficacy of CCX282-B in 436 patients with Crohn’s disease. Crohn’s Disease Activity Index (CDAI) scores were 250–450 and C-reactive protein >7.5 mg/L at study entry. In addition to stable concomitant Crohn’s medication (85% of subjects), subjects received placebo or CCX282-B (250 mg once daily, 250 mg twice daily, or 500 mg once daily) for 12 weeks. They then received 250 mg CCX282-B twice daily, open-label, through week 16. Subjects who had a clinical response (a ≥70 point drop in CDAI) at week 16 were randomly assigned to groups given placebo or CCX282-B (250 mg, twice daily) for 36 weeks. Primary endpoints were clinical response at Week 8 and sustained clinical response at Week 52. During the 12-week Induction period, the clinical response was highest in the group given 500 mg CCX282-B once daily. Response rates at week 8 were 49% in the placebo group, 52% in the group given CCX282-B 250 mg once daily (odds ratio [OR] = 1.12; p = .667 vs placebo), 48% in the group given CCX282-B 250 mg twice daily (OR = 0.95; p = .833), and 60% in the group given CCX282-B 500 mg once daily (OR = 1.53; p = .111). At week 12, response rates were 47%, 56% (OR = 1.44; p = .168), 49% (OR = 1.07; p = .792), and 61% (OR = 1.74; p = .039), respectively. At the end of the Maintenance period (week 52), 47% of subjects on CCX282-B were in remission, compared to 31% on placebo (OR = 2.01; p = .012); 46% showed sustained clinical responses, compared to 42% on placebo (OR = 1.14; p = .629). CCX282-B was well tolerated. Encouraging results from this clinical trial led to initiation of Phase 3 clinical trials in Crohn’s disease.

**Trial Registration:**

ClinicalTrials.gov NCT00306215.

## Introduction

Crohn’s disease is characterized by leukocyte infiltration of segments of the intestine, most commonly in the terminal ileum and colon, leading to mucosal erosion, ulceration, fistulization, and stenosis. [1] Chemokine receptors are G-protein coupled cell-surface proteins that interact with their chemokine ligands, which are low-molecular weight cytokine-like proteins, forming an elaborate system that regulates the migration and movement of inflammatory and immune cells within the body. [2] The C-C chemokine receptor CCR9 is expressed on a certain subset of circulating lymphocytes and is the principal chemokine receptor mediating homing to the intestinal mucosa, with enrichment of CCR9-positive cells in the intestine. [3] CCL25, or thymus-expressed chemokine (TECK), is the only identified CCR9 ligand [4], and is highly expressed in the intestine and thymus. CD8-positive T lymphocytes, plasmablasts, plasma cells, and plasmacytoid dendritic cells expressing CCR9 are involved in cellular interactions contributing to the pathogenesis of Crohn’s disease [5]–[8].

CCX282-B, also called Traficet-EN, GSK1605786A, or vercirnon, is a small molecule CCR9 antagonist that inhibits CCR9- and CCL25-dependent chemotaxis. Preclinical and early clinical studies suggested that orally-administered CCX282-B could reduce intestinal inflammation in inflammatory bowel disease (IBD). [9] This clinical trial, termed PROTECT-1 for Prospective Randomized Oral-Therapy Evaluation in Crohn’s disease, is the first major study to evaluate the safety and efficacy of a chemokine receptor antagonist in IBD.

## Methods

The protocol for this trial and supporting CONSORT checklist are available as supporting information; see Checklist S1 and Protocol S1.

### Ethics Statement

All subjects provided written informed consent prior to any study procedures. The names of ethics committees that reviewed and approved the clinical trial are provided in the Appendix.

All study procedures were governed by International Conference on Harmonisation Good Clinical Practice standards and the Declaration of Helsinki.

### Study Subjects

Ninety study centers in 17 countries in North America, Europe, Australia, Brazil, and South Africa enrolled and treated subjects from March 2006 to May 2009. The clinicaltrials.gov registration number is NCT00306215 (http://clinicaltrials.gov/ct2/show/NCT00306215?term=CCX282-B&rank=2). This clinical trial was sponsored by ChemoCentryx. Adult subjects with moderate to severe small bowel and/or colonic Crohn’s disease were enrolled. The Crohn’s Disease Activity Index (CDAI) [10] at screening was required to be 250 to 450, with fasting serum C-reactive protein (CRP) above 7.5 mg/L. Subjects receiving immunosuppressants or glucocorticoids (up to 20 mg prednisone-equivalent) had to be on stable doses for at least 4 weeks prior to randomization, and concomitant stable use of these drugs during the study was allowed. Concomitant 5-ASA treatment was also allowed. Anti-TNF or anti-α4 integrin treatment within 12 weeks prior to randomization was prohibited, and concomitant use during the study was not allowed.

### Study Design

This was a randomized, double-blind, placebo-controlled clinical trial to assess the efficacy and safety of CCX282-B in patients with moderate to severe Crohn’s disease. During the initial 12-week Induction period, subjects were randomized to receive placebo or CCX282-B, either 250 mg once daily (q.d.), 250 mg twice daily (b.i.d.), or 500 mg q.d. in a 1.5∶1∶1∶1 ratio. Randomization was performed centrally using an interactive voice response system. A blocked randomization schedule was generated by a biostatistician who was otherwise not involved in the study. Since efficacy in each CCX282-B group was compared to placebo, and for sample size efficiency, 1.5 times the number of subjects were randomized to the placebo group compared to each CCX282-B group. To conceal the allocation sequence, placebo and CCX282-B capsules, containing 250 mg CCX282-B, and bottles were identical in appearance and subjects received one kit (box) with three bottles of study medication every 4 weeks. The placebo kits contained three bottles of placebo capsules, the 250 mg q.d. kits contained one bottle of CCX282-B capsules (bottle 1) and two bottles of placebo (bottles 2 and 3), the 250 mg b.i.d. kits contained two bottles of CCX282-B capsules (bottles 1 and 3) and one bottle of placebo (bottle 2), and the 500 mg q.d. kits contained two bottles of CCX282-B capsules (bottles 1 and 2) and one bottle of placebo (bottle 3). Subjects were asked to take one capsule from each of the first two bottles every morning and one capsule from the third bottle every evening, 12 hours after the morning dose. Study medication was taken with water and could be taken with or without a meal.

The placebo-controlled portion of the study was double-blind. Blinding of the study was achieved by the following measures: (1) Study medication bottle and capsule appearances were identical; (2) Limited access was provided to the randomization code: Study site personnel, study subjects, personnel responsible for study monitoring, and biostatisticians and data managers involved in data analysis of the study, remained blinded to treatment assignment for the duration of the study; (3) While laboratory personnel conducting the pharmacokinetic (PK) assays were not blinded to treatment assignment, unblinded CCX282 plasma concentration results were not shared with the study site personnel or the study staff with direct contact with study sites during the study; (4) Efficacy data that could potentially be unblinding, such as serum CRP and CDAI scores, were not made available to study site personnel, study subjects, personnel responsible for study monitoring, and biostatisticians and data managers during the study unless required for safety monitoring.

After trial initiation, the protocol was amended to extend the study period from 12 weeks to 52 weeks to add a 4-week Active period and a 36-week Maintenance period. The amendment was implemented prior to having knowledge of the Induction period results. Subjects who had completed the Induction period of the study by the time of implementation of the protocol amendment, were allowed to enter the 4-week Active treatment period if they were within 3 months after completing the Induction period. All consenting subjects who completed 12 weeks in the Induction period received 250 mg CCX282-B b.i.d. from week 12 to 16 in an open-label Active period. This Active period was inserted between the Induction and Maintenance periods to allow all subjects, including those receiving placebo during the Induction period, to receive CCX282-B treatment and potentially to be included in the CDAI ≥70-point responder population for the Maintenance period. All consenting subjects who showed a clinical response at week 16 (CDAI decrease of ≥70 points compared to study baseline value), were re-randomized to receive placebo or CCX282-B 250 mg b.i.d., in a ratio of 1∶1.5 in the Maintenance period, from week 16 to 52. This change in the study allowed for evaluation of CCX282-B in both induction and maintenance settings. Corticosteroids, if used at week 16, were to be tapered to zero over a period not exceeding 6 weeks.

CDAI and CRP (high sensitivity assay with 3 mg/L as upper limit of normal) were recorded at baseline and weeks 4, 8, 12, 16, 20, 28, 36, 44, and 52. Consenting subjects, at study sites that agreed to participate, underwent colonoscopy at baseline and week 12. The Crohn’s Disease Endoscopic Index of Severity (CDEIS) was calculated using a modification of the method described by Mary and Modigliani. [11] The length of disease involvement and ulceration was recorded as actual lengths, in cm, rather than normalized on a 10-cm scale. CCX282 plasma concentrations were measured in samples taken at single random time points from all subjects at each study visit using high performance liquid chromatography with tandem mass spectrometric detection (lower limit of detection 2 ng/mL).

### Definition of Endpoints and Statistical Analysis

The primary efficacy endpoint for the Induction period was attainment of a clinical response, defined as a decrease in CDAI of at least 70 points from baseline, at week 8. Safety endpoints included the incidence of adverse events, serious adverse events, and withdrawals due to adverse events. Pre-defined secondary efficacy endpoints included clinical response at week 12, as well as clinical remission (CDAI ≤150) [12] and ≥100-point decrease in CDAI from baseline at weeks 8 and 12. The primary endpoint for the Maintenance period was maintenance of response from week 16 to week 52. Loss of response was defined as an increase in CDAI of more than 70 points and an increase to above 250, or a missing CDAI measurement. The main secondary endpoint was maintenance of remission through week 52. Loss of remission was defined as a CDAI above 150.

The primary analysis was in the intention-to-treat population for both Induction and Maintenance periods, including all randomized subjects who received study medication. Subjects who discontinued treatment, who had missing CDAI scores, or who received rescue treatment or surgery, were considered treatment failures from that point forward. For the primary and main pre-specified secondary endpoints, the statistical analysis was based on Mantel-Haenszel tests, stratified according to geographic region, comparing each CCX282-B group with the placebo group. Repeated measures mixed effect ANOVA models, including terms for treatment, time point, treatment by time point interaction, and geographic region were employed for continuous variables. All interactions were tested at an alpha level of 0.1 and statistical testing was two-sided.

Power calculations were based on a 25% placebo clinical response and a 50% clinical response in at least one CCX282-B group at week 8. A target number of 423 subjects would achieve 90% power at an overall alpha level of 0.05, 0.017 for each CCX282-B group compared to placebo, based on Bonferroni-adjusted Mantel-Haenszel testing. The protocol made allowance for an optional interim analysis when all subjects participating in the Maintenance period of the study had completed the Week 28 visit. A decision was made by the sponsor, upon review of the Induction period results, not to conduct this interim analysis, but to allow the study to run uninterrupted to completion. This decision was made to preserve the integrity of the Maintenance period of the trial. Therefore, the alpha level for the final analysis was 0.05.

## Results

### Study Subjects

**Figure 1:**
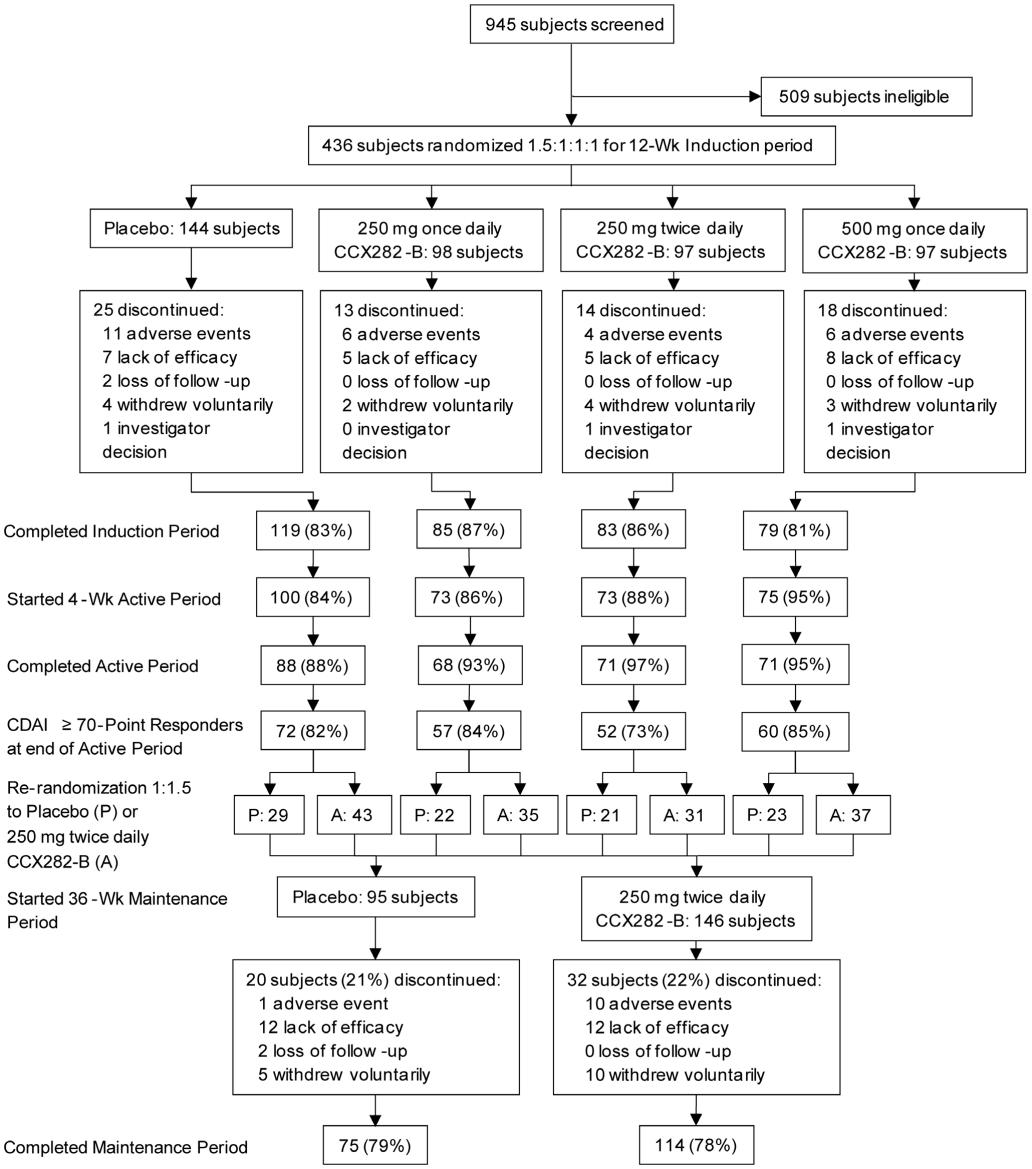
Subject Disposition. A total of 945 subjects were screened of whom 436 were randomized. Screen failure occurred most commonly for low CRP (57%) and low CDAI (24%). In the Induction period, 144 subjects were randomized to placebo, and 98, 97, and 97 to CCX282-B 250 mg q.d., 250 mg b.i.d., or 500 mg q.d., respectively. One subject, in the 250 mg b.i.d. group was excluded from the ITT population in the Induction period because the subject did not take any study medication. In the four groups, 83%, 87%, 86%, and 81% completed the Induction period. Most common primary reasons for withdrawal were adverse events and lack of efficacy with similar distributions across treatment groups. A total of 321 subjects started the 4-week open-label Active period, receiving CCX282-B 250 mg b.i.d.; 298 subjects completed this period, and of these, 241 clinical responders were enrolled in the Maintenance period. These subjects were re-randomized to placebo (95 subjects) or 250 mg b.i.d. CCX282-B (146 subjects). One subject, in the 250 mg b.i.d. group was excluded from the ITT population in the Maintenance period because the subject did not take any study medication. Of the two groups, 79% and 78%, respectively, completed the Maintenance period. The most common primary reason for withdrawal in the placebo group was lack of efficacy, 13% (12 of 95 subjects), compared to 8% (12 of 145 subjects) in the CCX282-B group, whereas the most common primary reason for withdrawal from the CCX282-B group was adverse events, 7% (10 of 145 subjects), compared to 1% (1 of 95 subjects) in the placebo group. Most of the adverse events leading to withdrawal in the CCX282-B group (9 of 10 cases) were related to Crohn’s disease. Arguably, withdrawal for lack of efficacy is similar to withdrawals because of Crohn’s disease-related adverse events. In both instances, the Crohn’s worsen or fail to improve. Therefore, when the number of withdrawals due to Crohn’s disease-related adverse events and lack of efficacy are added, the incidence is similar for the placebo and CCX282-B groups, i.e., 13/95 (14%) for placebo and 21/145 (14%) for CCX282-B.

Subject disposition is shown in Figure 1. Demographics and baseline characteristics of the study population were similar across treatment groups for the Induction period and the Maintenance period (Table 1). Of 945 subjects screened, 436 were randomized. Low CDAI and CRP were the main reasons for exclusion. Over 80% of subjects completed the Induction period, and nearly 80% the Maintenance period.

**Table 1:** Demographics and Baseline Characteristics of the Induction Period and Maintenance Period Intention-to-Treat Populations.

	Induction Period				Maintenance Period	
		CCX282-B Groups				
	Placebo(N = 144)	250 mg q.d.(N = 98)	250 mg b.i.d.(N = 96)	500 mg q.d.(N = 97)	Placebo(N = 95)	250 mg b.i.d.(N = 145)
Mean age–yr	36.6±12.24	37.2±11.69	37.3±13.24	34.9±12.14	34.6±12.63	37.2±11.77
Female–no. (%)	84 (58)	52 (53)	53 (55)	47 (49)	46 (48)	76 (52)
Mean body mass index–kg/m^2^	23.6±5.68	23.7±5.04	22.8±4.59	23.7±4.92	24.1±5.35	23.7±4.58
Current smoker–no. (%)	48 (33)	24 (25)	35 (37)	29 (30)	28 (30)	44 (30)
Median (range) duration of Crohn’s disease–yr	6 (0–31)	6 (0–33)	5 (0–25)	6 (0–44)	5 (0–35)	5 (0–26)
Median (range) of CDAI	321(250–454)	334(249–449)	327(249–471)	335(249–446)	136(3–371)	128(0–355)
Median (range) of CRP–mg per liter*	22(4–200)	22(3–124)	22(4–166)	21(7–182)	14(1–78)	13(0–97)
Location of Crohn’s disease–no. (%)						
Small intestine	117 (81)	83 (85)	83 (87)	76 (78)	81 (85)	118 (81)
Colon	117 (81)	81 (83)	72 (75)	71 (73)	71 (75)	111 (77)
Small intestine and colon	90 (63)	66 (67)	60 (63)	50 (52)	57 (60)	85 (59)
Rectum	67 (47)	38 (39)	38 (40)	38 (39)	37 (39)	67 (46)
Perianal	43 (30)	31 (32)	25 (26)	29 (30)	35 (37)	39 (27)
Previous bowel resection–no. (%)	30 (21)	29 (30)	24 (25)	26 (27)	26 (27)	29 (20)
Crohn’s medication use at start of study–no. (%)					–	–
none	24 (17)	14 (14)	10 (10)	16 (17)		
5-aminosalicylic acid	91 (63)	56 (57)	63 (66)	61 (63)		
azathioprine	41 (29)	32 (33)	27 (28)	22 (23)		
6-mercaptopurine	0	4 (4)	3 (3)	4 (4)		
methotrexate	7 (5)	4 (4)	4 (4)	2 (2)		
corticosteroids	55 (38)	38 (39)	40 (42)	33 (34)		
Previous biologic treatment–no. (%)					–	–
infliximab	32 (22)	28 (29)	25 (26)	23 (24)		
adalimumab	11 (8)	6 (6)	5 (5)	9 (9)		
natalizumab	10 (7)	3 (3)	5 (5)	5 (5)		
No. of subjects with baseline andweek 12 colonoscopies	37	16	21	16	–	–
Median (range) of CDEIS, per protocol	16.8 (2–58)	25.5 (3–65)	14.0 (1–48)	23.0 (3–78)	–	–
Median (range) of CDEIS, as definedin ref. 11	9.7 (1–24)	12.4 (3–28)	7.8 (0–19)	11.3 (3–32)	–	–

* A total of 8 subjects (2, 3, 2, and 1 in the placebo, 250 mg q.d., 250 mg b.i.d., and 500 mg q.d. groups, respectively) had baseline CRP values ≤ 7.5 mg/L, a deviation from the study protocol inclusion criteria.

### Efficacy

**Figure 2:**
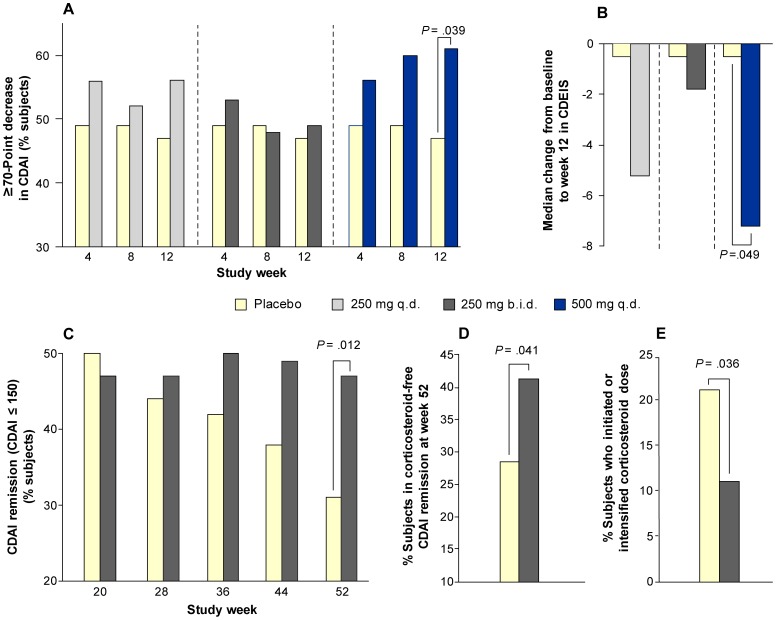
Clinical Efficacy Results. The percentage of subjects who had a CDAI decrease of at least 70 points is shown at each Induction period study visit after the baseline (A). Placebo n = 144, CCX282-B 250 mg q.d. n = 98, CCX282-B 250 mg b.i.d. n = 96, CCX282-B 500 mg q.d. n = 97; (B) The median change from baseline to week 12 in CDEIS, calculated per protocol (Placebo n = 37, CCX282-B 250 mg q.d. n = 16, CCX282-B 250 mg b.i.d. n = 21, CCX282-B 500 mg q.d. n = 16); (C) The percentage of subjects in each treatment group who were in remission (CDAI ≤150) at each visit over the course of the Maintenance period (Placebo n = 95 and CCX282-B 250 mg b.i.d. n = 145); (D) The percentage of subjects by treatment group who achieved corticosteroid-free remission, i.e., CDAI ≤150, at the end of the Maintenance period of the clinical trial, and (E) The percentage of subjects by treatment group who required an increase in corticosteroid dose or initiation of new corticosteroid use during the Maintenance period of the clinical trial.

Subjects underwent an aggregate treatment regimen of 52 weeks: a 12-week Induction period followed by a 4-week open-label Active period and a 36-week Maintenance period. Table 2 shows the CDAI response and remission results from the 12-week Induction period. The CCX282-B 500 mg q.d. group showed the highest CDAI response rates (≥70 point decrease in CDAI): 56%, 60%, and 61% at weeks 4, 8, and 12, respectively (Table 2 and Figure 2a). The placebo group response rates were 49%, 49%, and 47%, respectively, at these time points. The odds ratio (OR) between 500 mg q.d. CCX282-B and placebo was 1.53; 98.3% Confidence Interval [CI] 0.81, 2.89; p = 0.111 at Week 8, the predefined primary time point, and the OR was 1.74; 98.3% CI 0.92, 3.29; p = 0.039 at week 12, the predefined secondary time point. The 250 mg CCX282-B groups showed no significant differences in CDAI response compared to placebo at any time point. The 500 mg q.d. group showed higher clinical response rates compared with placebo and the 250 mg groups across all gender, Crohn’s disease anatomic location strata, including colonic Crohn’s disease, as well as other concomitant Crohn’s medication use strata (Table 3). Age, race, gender, disease location, and Crohn’s medication did not show significant interaction (at p<0.1) with CDAI response. During the Induction period, the CDAI remission (≤150) rate was not significantly different across treatment groups (Table 2). The proportion of subjects with ≥100 point decrease in CDAI was 41%, 49%, and 55%, at weeks 4, 8, and 12, respectively, in the 500 mg q.d. group compared to 40%, 42%, and 40%, respectively, for the placebo group (Table 2); the OR between 500 mg CCX282-B q.d. and placebo at Week 12 was 1.79; 98.3% CI 0.95, 3.37; p = 0.029. The 250 mg groups did not show significant differences compared to placebo.

**Table 2:** Clinical Response and Remission Results During the 12-Week Induction Period.

	Placebo(N = 144)	250 mg q.d. CCX282-B(N = 98)	250 mg b.i.d. CCX282-B(N = 96)	500 mg q.d. CCX282-B(N = 97)
**Clinical response** a				
Week 4, n (%)^b^	70 (48.6)	55 (56.1)	51 (53.1)	54 (55.7)
Week 8, n (%)	71 (49.3)	51 (52.0)	46 (47.9)	58 (59.8)
Week 12, n (%)	68 (47.2)	55 (56.1)	47 (49.0)	59 (60.8)*
**Clinical remission** c				
Week 4, n (%)	21 (14.6)	16 (16.3)	13 (13.5)	15 (15.5)
Week 8, n (%)	37 (25.7)	23 (23.5)	16 (16.7)	23 (23.7)
Week 12, n (%)	39 (27.1)	25 (25.5)	21 (21.9)	29 (29.9)
**CDAI decrease from baseline ≥100**				
Week 4, n (%)	58 (40.3)	40 (40.8)	40 (41.7)	40 (41.2)
Week 8, n (%)	61 (42.4)	40 (40.8)	42 (43.8)	47 (48.5)
Week 12, n (%)	58 (40.3)	47 (48.0)	40 (41.7)	53 (54.6)**

a Clinical response is defined as a decrease in CDAI score from baseline ≥70.

b n (%) = number and percentage of subjects in each group; % calculated as n/N x 100.

c Clinical remission is defined as a CDAI score ≤150.

* p = 0.039; p-value was obtained from Mantel-Haenszel test, stratified according to geographic region, comparing each CCX282-B group with the placebo group.

** p = 0.029; p-value was obtained from Mantel-Haenszel test, stratified according to geographic region, comparing each CCX282-B group with the placebo group.

**Table 3:** CDAI Clinical Response at Week 12 by Gender, Crohn’s Disease Location, and Other Concomitant Crohn’s Medication Use.

	Placebo(N = 144)	250 mg q.d. CCX282-B(N = 98)	250 mg b.i.d. CCX282-B(N = 96)	500 mg q.d. CCX282-B(N = 97)
**Gender**				
Male				
N’	60	46	43	50
n (%)	27 (45.0%)	27 (58.7%)	20 (46.5%)	33 (66.0%)*
Female				
N’	84	52	53	47
n (%)	40 (47.6%)	28 (53.8%)	27 (50.9%)	26 (55.3%)
**Crohn’s Disease Location**				
Small intestine				
N’	117	83	83	76
n (%)	56 (47.9%)	46 (55.4%)	41 (49.4%)	47 (61.8%)#
Colon				
N’	117	81	72	71
n (%)	58 (49.6%)	45 (55.6%)	33 (45.8%)	44 (62.0%)
Small intestine and colon				
N’	90	66	60	50
n (%)	46 (51.1%)	36 (54.5%)	28 (46.7%)	32 (64.0%)
Rectum and perianal				
N’	28	19	12	20
n (%)	13 (46.4%)	12 (63.2%)	6 (50.0%)	15 (75.0%)#
**Other Concomitant Crohn’s Medication Use** a				
Users				
N’	111	86	81	75
n (%)	52 (46.8%)	50 (58.1%)	42 (51.9%)	45 (60.0%)#
Non-Users				
N’	33	12	15	22
n (%)	16 (48.5%)	5 (41.7%)	5 (33.3%)	14 (63.6%)

N’ = number of subjects in the ITT population for each subgroup; % = n/N’ x 100.

a Other concomitant Crohn’s medications include 5-ASA, corticosteroids, 6-mercaptopurine, azathioprine, and methotrexate.

* p<0.05 for comparison with placebo based on Fisher’s exact test.

#p<0.10 for comparison with placebo based on Fisher’s exact test.

Approximately 26% of subjects (112 of 435) had previously received anti-TNF therapies, and 53 of these were non-responsive to one or more of these anti-TNF therapies based on data collected during screening. CDAI results from an exploratory analysis in the anti-TNF-experienced subjects are shown in Table S1. In the subset of subjects who were non-responsive to one or more anti-TNF drugs, CDAI ≥70 and ≥100 point responses at week 12 occurred in 8 of 14 (57%) subjects on CCX282-B 500 mg q.d., compared to 5 of 18 (28%) on placebo. Remission was observed in 3 of 14 (21%) on CCX282-B 500 mg q.d. and 1 of 18 (6%) on placebo.

The 321 subjects who entered the Active period, during which all subjects received CCX282-B 250 mg b.i.d., had a median CDAI of 184. At the end of the 4-week Active period, the median CDAI was 160. Of subjects completing the Active period, 241 (75.1%) showed a clinical response with a drop in CDAI of ≥70 (relative to their baseline value), and were re-randomized in a 1∶1.5 ratio to receive placebo or CCX282-B 250 mg b.i.d. in the 36-week Maintenance period.

CDAI response and remission results for the Maintenance period are shown in Figure 2 and elaborated in Table 4. At week 52, 47% of subjects on CCX282-B were in remission compared with 31% on placebo (OR 2.01; 95% CI 1.16, 3.49; p = 0.012; Figure 2c). In a pre-specified last-observation-carried-forward sensitivity analysis, remission was achieved in 74 of 145 subjects (51%) in the CCX282-B group and 32 of 95 subjects (34%) in the placebo group (OR 2.05, 95% CI 1.20, 3.51; p = 0.009). Furthermore, at week 52, 41% (60 of 145 subjects) on CCX282-B were in corticosteroid-free remission compared to 28% (27 of 95 subjects) on placebo (p = 0.041; see Figure 2d). A sustained response at every visit during the Maintenance period was seen in 46% of subjects in the CCX282-B 250 mg b.i.d. group compared to 42% in the placebo group (OR 1.14, 95% CI 0.67, 1.92; p = 0.629), and sustained remission (CDAI ≤150) was observed in 41% in the CCX282-B group and 30% in the placebo group (OR 1.60, 95% CI 0.77, 3.30; p = 0.205). In a pre-specified last-observation-carried-forward sensitivity analysis, sustained remission was achieved in 42 of 87 subjects (48%) in the CCX282-B group and 17 of 53 subjects (32%) in the placebo group (OR 1.94, 95% CI 0.95, 3.95; p = 0.066).

**Table 4:** Clinical Response and Remission Results for the 36-Week Maintenance Period.

	Placebo(N = 95)	250 mg b.i.d. CCX282-B(N = 145)	Placebo(N = 53)	250 mg b.i.d. CCX282-B(N = 87)	Placebo(N = 95)	250 mg b.i.d. CCX282-B(N = 145)
	Sustained response^a^		Sustained remission^b^		Remission at each time point	
Week 16, n (%)^c^	95 (100.0%)	145 (100.0%)	53 (100.0%)	87 (100.0%)	53 (55.8%)	88 (60.7%)
Week 20, n (%)	75 (78.9%)	118 (81.4%)	39 (73.6%)	59 (67.8%)	47 (49.5%)	68 (46.9%)
Week 28, n (%)	62 (65.3%)	93 (64.1%)	28 (52.8%)	47 (54.0%)	42 (44.2%)	68 (46.9%)
Week 36, n (%)	54 (56.8%)	82 (56.6%)	23 (43.4%)	47 (54.0%)	40 (42.1%)	73 (50.3%)
Week 44, n (%)	48 (50.5%)	75 (51.7%)	20 (37.7%)	43 (49.4%)	36 (37.9%)	71 (49.0%)
Week 52, n (%)	40 (42.1%)	66 (45.5%)	16 (30.2%)	36 (41.4%)	29 (30.5%)	68 (46.9%)*

a Sustained response was defined as a decrease in CDAI score from baseline (last non missing value before the first dose in the Induction period) ≥70 at Week 16 and no loss of the response during the 36-week Maintenance period. Loss of clinical response was defined as a CDAI score increase at any visit after the Week 16 visit of ≥70 from the Week 16 value and an absolute CDAI value of >250, or the need for intervention after Week 16. Missing values were also imputed as loss of response.

b Sustained remission was defined as a CDAI score ≤150 at Week 16 and Week 52 and all visits in between. If data from any time point between Week 16 and 62 were missing for a subject, sustained remission was not achieved.

c n (%) = number and percentage of subjects in each group; % calculated as n/N x 100.

* p = 0.012; p-value was obtained from Mantel-Haenszel test, stratified according to geographic region, comparing the CCX282-B group with the placebo group.

The changes from baseline to week 12 in CDAI, CRP, and CDEIS are shown in Table 5. The CCX282-B 500 mg q.d. group showed the largest mean decreases from baseline to week 12 in CDAI, CRP, and CDEIS. Mean change from baseline to week 12 in CDEIS was −3.0 in placebo, compared with −8.7, 2.0, and −10.8 (p = 0.049 vs. placebo; Figure 2b) in the CCX282-B groups, respectively. Mean change from baseline to week 12 in CDEIS, calculated using normalized lengths of Crohn’s involvement [11], was −1.1 in placebo, compared with −3.1, 0.9, and −4.4 (p = 0.049 vs. placebo) in the CCX282-B groups, respectively.

**Table 5:** Change from Baseline to Week 12 in CDAI, CRP, and CDEIS.

	Placebo(N = 144)	250 mg q.d. CCX282-B(N = 98)	250 mg b.i.d. CCX282-B(N = 96)	500 mg q.d. CCX282-B(N = 97)
**Crohn’s Disease Activity Index**				
Baseline				
Mean (SD)	329.9 (49.47)	340.1 (54.29)	332.3 (52.36)	332.5 (55.68)
Median	321.0	333.5	326.5	335.0
Min, Max	250, 454	249, 449	249, 471	249, 446
n	144	98	96	97
Week 12–Change from baseline				
Mean (SD)	−115.9 (116.07)	−134.2 (99.87)	−121.7 (101.21)	−136.0 (102.05)
Median	−126.9	−147.1	−125.3	−146.2
Min, Max	−368, 251	−345, 88	−375, 105	−338, 109
n	120	85	85	87
**C-reactive protein, mg/L**				
Baseline				
Mean (SD)	30.8 (27.87)	28.0 (22.62)	27.6 (22.63)	30.3 (27.13)
Median	21.6	22.3	21.8	21.4
Min, Max	4.4, 200.0	2.7, 124.0	4.3, 165.5	6.6, 182.0
n	144	98	96	97
Week 12–Change from baseline				
Mean (SD)	−4.4 (34.08)	−4.1 (26.50)	−1.4 (31.06)	−6.6 (34.84)
Median	−2.9	−3.1	−4.5	−6.7
Min, Max	−199.6, 115.2	−123.7, 72.1	−82.6, 133.6	−175.4, 117.0
N	138	97	96	94
**Crohn’s Disease Endoscopic Index of Severity (CDEIS)** a				
Baseline				
Mean (SD)	19.1 (15.02)	23.5 (20.56)	18.7 (14.14)	29.5 (23.28)
Median	14.0	16.2	16.5	23.0
Min, Max	2, 58	1, 65	1, 48	2, 78
n	48	24	26	22
Week 12–Change from baseline				
Mean (SD)	−3.0 (11.44)	−8.7 (11.60)	2.0 (10.75)	−10.8 (17.70)*
Median	−0.5	−5.2	−1.8	−7.2
Min, Max	−28, 30	−30, 6	−12, 35	−51, 17
n	37	16	21	16

a CDEIS calculated using a modification of the method described by Mary and Modigliani^13^. The length of disease involvement and ulceration was recorded as actual lengths, in cm, rather than normalized on a 10-cm scale.

* p = 0.049; p-value based on a repeated measures mixed effect ANOVA model, including terms for treatment, time point, treatment by time point interaction, and geographic region.

During the 36-week Maintenance period, median CDAI decreased from 128 to 95 in the CCX282-B group and increased from 136 to 146 in the placebo group. Median CRP levels decreased from 12.6 mg/L to 8.7 mg/L in the CCX282-B group, and from 14.1 mg/L to 12.3 mg/L in the placebo group from week 16 to 52. Systemic corticosteroid therapy was initiated or intensified in 21% (20/95 subjects) of the placebo group and 11% (16/145 subjects) of the CCX282-B group (p = 0.036; see Figure 2e). Conversely, corticosteroid therapy in CCX282-B subjects was stopped in 57% (32/56 subjects on corticosteroids at the start of the Maintenance period) compared to 43% (13/30 subjects) in the placebo group. Anti-TNF rescue treatment was given to 4% (4 of 95 subjects) in the placebo group and 1% (1 of 145 subjects) in the CCX282-B group. One subject, withdrawn from CCX282-B treatment, received natalizumab during the Maintenance period.

Population pharmacokinetic data from the Induction period indicated that the highest maximum plasma concentration (C_max_) occurred with 500 mg q.d. CCX282-B. The modeled estimated mean (±SD) C_max_ was 736 (±141), 812 (±147), and 1032 (±163) ng/mL in the CCX282-B 250 mg q.d., 250 mg b.i.d., and 500 mg q.d. groups, respectively.

### Safety

Safety results are summarized in Table 6. In the Induction period, 63% (90 subjects) on placebo reported at least one adverse event compared to 60% (174 subjects) on CCX282-B. The incidence of the most common adverse events was similar across treatment groups. Serious adverse events occurred in 10% placebo versus 9% in the CCX282-B group (15/144 subjects compared to 25/291, respectively). Study drug withdrawals due to adverse events in the CCX282-B group occurred in 7% (20 subjects) compared to 13% (19 subjects) in the placebo group. A lower incidence of withdrawals attributed to gastrointestinal adverse events and exacerbation of Crohn’s disease was also observed in the CCX282-B group overall compared to placebo. One subject, in the placebo group, died during the study from septic complications following intestinal perforation. Another subject, in the 250 mg b.i.d. group, who had lack of response after the week 16 visit, died 39 days later due to complications of Crohn’s disease that were deemed unrelated to study medication. Infections occurred in 16% of both placebo and CCX282-B groups during the Induction period (23/144 on placebo and 47/291 on CCX282-B). No opportunistic infections were reported. No clinically significant changes in laboratory parameters occurred in any of the treatment groups.

**Table 6:** Adverse Events in the Safety Population.

	Induction Period					Active Period	Maintenance Period	
		CCX282-B						
**Event**	**Placebo** **(N = 144)**	**250 mg q.d.** **(N = 98)**	**250 mg b.i.d.** **(N = 96)**	**500 mg q.d.** **(N = 97)**	**All** **(N = 291)**	**250 mg b.i.d. CCX282-B** **(N = 318)**	**Placebo** **(N = 95)**	**250 mg b.i.d. CCX282-B** **(N = 145)**
	*No. of subjects (%)*							
Any event	90 (63)	62 (64)	56 (58)	56 (58)	174 (60)	88 (28)	58 (61)	98 (68)
Event in ≥5% of placebo or CCX282-B group overall								
Abdominal pain	19 (13)	16 (17)	18 (19)	12 (12)	46 (16)	14 (4)	19 (20)	19 (13)
Crohn’s disease	10 (7)	9 (9)	14 (14)	6 (6)	29 (10)	12 (4)	7 (7)	17 (12)
Diarrhoea	11 (8)	10 (10)	7 (7)	7 (7)	24 (8)	12 (4)	14 (15)	15 (10)
Nausea	10 (7)	8 (8)	10 (10)	6 (6)	24 (8)	4 (1)	7 (7)	6 (4)
Dyspepsia	5 (4)	7 (7)	3 (3)	9 (9)	19 (7)	1 (0.3)	0	5 (3)
Headache	7 (5)	6 (6)	8 (8)	5 (5)	19 (7)	2 (1)	3 (3)	3 (2)
Arthralgia	8 (6)	10 (10)	3 (3)	3 (3)	16 (6)	3 (1)	7 (7)	14 (10)
Pyrexia	8 (6)	5 (5)	4 (4)	5 (5)	14 (5)	8 (3)	2 (2)	7 (5)
Abdominal tenderness	3 (2)	3 (3)	5 (5)	4 (4)	12 (4)	1 (0.3)	6 (6)	4 (3)
Vomiting	5 (4)	4 (4)	5 (5)	3 (3)	12 (4)	8 (3)	2 (2)	8 (6)
Serious adverse events	15 (10)	5 (5)	11 (11)	9 (9)	25 (9)	12 (4)	9 (10)	13 (9)
Events leading to study treatment withdrawal	19 (13)	8 (8)	5 (5)	7 (7)	20 (7)	9 (3)	6 (6)	10 (7)
Gastrointestinal events leading to study treatment withdrawal	14 (10)	6 (6)	3 (3)	5 (5)	14 (5)	9 (3)	4 (4)	9 (6)
Crohn’s disease leading to study treatment withdrawal	6 (4)	3 (3)	0	1 (1)	4 (1)	5 (2)	1 (1)	4 (3)
Adverse events leading to death	1 (1)	0	0	0	0	1 (0.3)^a^	0	0
Infection or infestation	23 (16)	11 (11)	18 (19)	18 (19)	47 (16)	20 (6)	19 (20)	35 (24)
Transaminase increase >3× upper limit	1 (1)	0	1 (1)	0	1 (0.3)	0	1 (1)	1 (0.7)

a Event occurred 39 days after study discontinuation.

The incidence of the most common adverse events during the Maintenance period was similar across treatment groups, and 58 of 95 subjects (61%) in the placebo group reported at least one adverse event compared to 98 of 145 subjects (68%) in the CCX282-B 250 mg b.i.d. group. Serious adverse events occurred in 10% of placebo group versus 9% in the CCX282-B group (9/95 subjects versus 13/145, respectively). No serious infections or opportunistic infections were observed during the Maintenance period and no deaths occurred.

## Discussion

CCX282-B, an orally-administered CCR9 antagonist, is the first chemokine receptor antagonist to be tested in IBD. In this clinical trial, the efficacy, safety and tolerability of CCX282-B were evaluated in a 12-week Induction period, followed by a 4-week Active period, and then a 36-week Maintenance period. Blocking CCR9 is a new approach to Crohn’s disease therapy and a test of this mechanism has not been attempted previously in clinical trials. Accordingly, the trial design largely reflected that of other agents, predominantly the anti-TNF modalities. While primary endpoints of this trial were not achieved, pre-specified secondary endpoints were met.

Patients with both small and large bowel disease were eligible for the trial. The decision to include patients with large bowel disease was based on experimental evidence that CCR9 and TECK are expressed in both small and large bowel[13]–[15], and emerging experimental evidence that CCR9 inhibition is effective in the MDR1a^−/−^ mouse model of colitis [15].

In the Induction period of the study, the CCX282-B 500 mg q.d. group showed the highest CDAI response. The difference in clinical response rate (drop in CDAI ≥70 points) between 500 mg q.d. CCX282-B and placebo (ΔCDAI) was 7%, 11%, and 14% at weeks 4, 8, and 12, respectively. This increasing ΔCDAI was also observed for CDAI ≥100-point response (1%, 6%, and 14%, respectively). This suggests that the full effect of CCX282-B may take longer than 8 weeks to manifest clinically, and week 12 might have been a more appropriate primary time point for the Induction period.

The ΔCDAI of 14% at week 12 for CDAI ≥70-point response between 500 mg q.d. CCX282-B and placebo is generally comparable to results from clinical trials of other therapies that have found a place in clinical practice. For example, at 12 weeks, the ΔCDAI was 10 to 16% with natalizumab [16], [17] and 9% with certolizumab. [18] There are no 12-week time point data available for adalimumab [19].

CDAI clinical response differences between 500 mg q.d. CCX282-B and placebo were seen across all GI location strata (Table 3); the treatment difference was most pronounced in patients with rectal and perianal disease (ΔCDAI 29%). There are insufficient patient numbers in each GI location stratum to be definitive, but this finding, if confirmed, would support a role for CCX282-B in treatment of patients with both small and large bowel disease.

Differences in the pharmacokinetic profiles between the 500 mg and 250 mg dosage regimens might explain the apparent differences in efficacy results. Chemotaxis studies of human lymphocytes in 100% human serum indicate that 800 ng/mL CCX282 is required to block 90% of CCR9 receptors (serum IC_90_). Furthermore, full efficacy in pre-clinical studies required CCX282 plasma concentrations above the serum IC_90_. [9] Based on modeled population pharmacokinetic profiles, plasma concentrations with 500 mg q.d. CCX282-B exceeded the serum IC_90_ for at least 8 hours following dosing. However, the mean maximum CCX282 plasma concentrations at the 250 mg b.i.d dose were probably sub-therapeutic in the Induction setting. This difference in CCX282 plasma exposure between the 500 mg q.d. and 250 mg b.i.d. dosing regimens may explain the different clinical efficacy profiles in the Induction period. Since plasma samples were taken only at single time points at study visits, sparse data were available for each subject, and full pharmacokinetic profiles are not available for individual subjects. Therefore, correlations of individual pharmacokinetic parameters such as C_max_ or AUC with CDAI response could not be made reliably. CCX282-B 250 mg b.i.d. was selected as the dose for the Active and Maintenance periods before data from the Induction period, showing that 500 mg q.d. might be more effective, were available. Although the 250 mg b.i.d. dose was apparently ineffective in the Induction period, this dose provided some evidence of efficacy in the Maintenance period. One potential explanation for these observations is that higher CCX282 plasma concentrations may be required to induce a clinical response in active disease, while lower plasma concentrations may effectively sustain remission subsequently when disease activity is lower. Such findings are not uncommon for maintenance dosing during remission with a variety of therapeutic modalities in chronic disease.

Changes in CRP and CDEIS support the efficacy of 500 mg q.d. CCX282-B in the Induction period. Of all four groups, 500 mg q.d. CCX282-B showed the largest decrease in serum CRP, of approximately 30%, at 12 weeks. The higher CRP level entry criterion of >7.5 mg/L in PROTECT-1 compared to other clinical trials, which resulted in a higher mean baseline CRP compared to these trials, [16], [18], [19] limits comparison across studies. Nonetheless, the absolute decrease in CRP with 500 mg CCX282-B is generally comparable to the changes observed with anti-TNF agents [18], [19] and with natalizumab. [17] Subjects receiving 500 mg q.d. CCX282-B also showed a greater decrease in CDEIS compared to placebo, suggesting that CCX282-B treatment may improve intestinal mucosal lesions.

The percentage of subjects reaching clinical remission (CDAI of ≤150), was significantly higher in the CCX282-B group compared to placebo at the end of the Maintenance period, even though remission rates were not significantly different among groups in the Induction period. The baseline CDAI of the study population in PROTECT-1 was 333 (±53), or approximately 30 points higher than in other recent studies, [16], [18], [19] so that a larger absolute drop in CDAI was required to reach the CDAI score of 150, which was used to define remission. This high baseline CDAI could therefore have reduced the chance of achieving remission in a 12-week Induction trial, and of demonstrating a difference between CCX282-B and placebo groups. Some support for this contention was provided by an exploratory analysis indicating that with CDAI “near-remission” 5-point incremental thresholds of 155 up to 170, the near-remission rate in the CCX282-B 500 mg q.d. group was up to 10% higher than the placebo group (38.1% compared to 28.5% with 170 as threshold; Table S2).

In the Maintenance period, the incidence of relapse in the placebo group was 58% over 36 weeks, which was lower than anticipated. In a comparable natalizumab clinical trial, the incidence of relapse was 72% at the 36-week time point in the placebo group. [16] Therefore, the placebo response rate in our study was higher than expected. A possible reason is that the majority of subjects in this study (85%), including the subjects receiving placebo, were receiving background medications for Crohn’s disease including corticosteroids. This difference may also reflect variations in clinical trial design. For example, in PROTECT-1, all subjects received 4 weeks of CCX282-B treatment at the end of the Induction period and before entering the Maintenance period. In the natalizumab clinical trial, [16] there was no active treatment period between the Induction period and the Maintenance period.

Although subjects who have previously received anti-TNF agents often have lower response rates to other treatments, in this study, exploratory analyses showed that CCX282-B might have utility in these subjects. This finding is a topic of further study in ongoing Phase 3 clinical trials.

The apparent safety and tolerability of CCX282-B observed in this study are encouraging as most other medications for Crohn’s disease have limitations such as increased risk of infection. Thiopurines, widely used to maintain remission, are also associated with an increased risk of neoplasia, particularly of the lymphohemopoietic system. [20] Preclinical and early phase CCX282-B clinical studies have supported the favorable safety and tolerability profile, [9] and if confirmed in larger studies, would be beneficial.

Limitations of this study include the relatively small sample size, the strict inclusion criteria, defining patients with moderately to severely active Crohn’s disease, so that results cannot yet be generalized to less or more severely affected patients. Furthermore, as this is the first major trial of a CCR9 antagonist, the design of the trial and selection of time points relied on evidence from mechanistically unrelated medical treatments. Lastly, the placebo response rates observed during both the Induction and Maintenance periods of this trial were relatively high.

In conclusion, this study suggests that CCX282-B, an orally-administered and specific CCR9 antagonist, provides a novel approach to modulating intestine-specific inflammatory responses, and if results are confirmed might be an effective therapy for IBD. Targeting the chemokine system offers the prospect of organ specific immunotherapy for a wide variety of immunologically-activated diseases. Phase 3 clinical trials are now being conducted to further evaluate the safety and efficacy of CCX282-B (also called GSK1605786A or vercirnon) in groups of patients with broader inclusion criteria.

## Supporting Information

Table S1 **CDAI Results in Anti-TNF Users.** A total of 112 patients previously used anti-TNF drugs, adalimumab or infliximab, for treatment of their Crohn’s disease. The number and percentage of patients who had a decrease of at least 70 points in CDAI, CDAI ≤150 (remission), and a decrease of at least 100 points in CDAI at week 12 in the clinical trial are shown by treatment group in this table.
(DOCX)

Table S2 **CDAI “Near Remission” Results.** The number and percentage of patients who reached CDAI thresholds of ≤155, ≤160, ≤165, and ≤170 at week 12 in the clinical trial are shown in this table.
(DOCX)

Checklist S1 **CONSORT Checklist.**
(DOC)

Protocol S1 **Study Protocol.**
(DOC)

Appendix S1 **Study Centers and Ethics Committees.**
(DOC)
