# Supplementary material for: A Randomized Controlled Trial of the Efficacy and Safety of CCX282-B, an Orally-Administered Blocker of Chemokine Receptor CCR9, for Patients with Crohn’s Disease
Source: PLoS One. 2013 Mar 20;8(3):e60094. doi: 10.1371/journal.pone.0060094 (PMC3603920; doi:10.1371/journal.pone.0060094)
Supplement: Table S1 — CDAI Results in Anti-TNF Users. A total of 112 patients previously used anti-TNF drugs, adalimumab or infliximab, for treatment of their Crohn’s disease. The number and percentage of patients who had a decrease of at least 70 points in CDAI, CDAI ≤150 (remission), and a decrease of at least 100 points in CDAI at week 12 in the clinical trial are shown by treatment group in this table. (DOCX) [file pone.0060094.s001.docx]

Table S1. CDAI Response and Remission at Week 12 in Patients who Previously Received Anti-TNF Drugs Including Infliximab and Adalimumab

|  | **Placebo**  **(N=35)** | **250 mg q.d. CCX282-B**  **(N=29)** | **250 mg b.i.d. CCX282-B**  **(N=25)** | **500 mg q.d. CCX282-B**  **(N=23)** |
| --- | --- | --- | --- | --- |
| CDAI decrease from baseline ≥ 70 points | | | | |
| n (%) | 10 (28.6%) | 10 (34.5%) | 8 (32.0%) | 12 (52.2%) |
| CDAI ≤ 150 | | | | |
| n (%) | 4 (11.4%) | 6 (20.7%) | 5 (20.0%) | 4 (17.4%) |
| CDAI decrease from baseline ≥ 100 points | | | | |
| n (%) | 10 (28.6%) | 9 (31.0%) | 7 (28.0%) | 11 (47.8%) |
