# Supplementary material for: A Randomized Controlled Trial of the Efficacy and Safety of CCX282-B, an Orally-Administered Blocker of Chemokine Receptor CCR9, for Patients with Crohn’s Disease
Source: PLoS One. 2013 Mar 20;8(3):e60094. doi: 10.1371/journal.pone.0060094 (PMC3603920; doi:10.1371/journal.pone.0060094)
Supplement: Table S2 — CDAI “Near Remission” Results. The number and percentage of patients who reached CDAI thresholds of ≤155, ≤160, ≤165, and ≤170 at week 12 in the clinical trial are shown in this table. (DOCX) [file pone.0060094.s002.docx]

Table S2. CDAI “Near Remission” Responses at Week 12 Based on Different CDAI Thresholds

| **Week 12 CDAI Thresholds for Response** | **Placebo**  **(N=144)** | **250 mg q.d. CCX282-B**  **(N=98)** | **250 mg b.i.d. CCX282-B**  **(N=96)** | **500 mg q.d. CCX282-B**  **(N=97)** |
| --- | --- | --- | --- | --- |
| CDAI ≤ 155 | 39 (27.1%) | 25 (25.5%) | 25 (26.0%) | 31 (32.0%) |
| CDAI ≤ 160 | 41 (28.5%) | 25 (25.5%) | 25 (26.0%) | 34 (35.1%) |
| CDAI ≤ 165 | 41 (28.5%) | 25 (25.5%) | 25 (26.0%) | 36 (37.1%) |
| CDAI ≤ 170 | 41 (28.5%) | 26 (26.5%) | 28 (29.2%) | 37 (38.1%) |
