# Supplementary material for: A Randomized Controlled Trial of the Efficacy and Safety of CCX282-B, an Orally-Administered Blocker of Chemokine Receptor CCR9, for Patients with Crohn’s Disease
Source: PLoS One. 2013 Mar 20;8(3):e60094. doi: 10.1371/journal.pone.0060094 (PMC3603920; doi:10.1371/journal.pone.0060094)
Supplement: Protocol S1 — Study Protocol. (DOC) [file pone.0060094.s004.doc]

# Protocol S1

# PROTOCOL

# TITLE PAGE

| **Study Title:** | A Multinational Double-Blind, Placebo-Controlled, Parallel Group Study to Evaluate the Efficacy and Safety of CCX282-B in Subjects with Moderate to Severe Crohn’s Disease |
| --- | --- |
| **Protocol Number:** | CL004_282 |
| **Investigational Product:** | Chemokine receptor 9 antagonist CCX282-B |
| **Indication:** | Inflammatory Bowel Disease / Crohn’s Disease |
| **Sponsor:** | ChemoCentryx, Inc. |

| Name of Sponsor  ChemoCentryx, Inc | Name of Active Ingredient  CCX282-B | Study number  CL004_282 |
| --- | --- | --- |
| **Title of Study:** A Multinational Double-Blind, Placebo-Controlled, Parallel Group Study to Evaluate the Efficacy and Safety of CCX282-B in Subjects with Moderate to Severe Crohn’s Disease | | |
| **Investigator:** Multicenter | | |
| **Study Center:** Up to approximately 100 centers | | |
| **Study Period:** Approximately 24 months | | |
| **Objectives:** The primary efficacy objective of the induction period (first 12 weeks) of this study is to determine the effect on Crohn’s disease severity as measured by Crohn’s Disease Activity Index (CDAI) scores of three dose regimens of CCX282-B compared with placebo, administered orally for up to twelve consecutive weeks to subjects with moderate to severe Crohn’s disease. Secondary efficacy objectives of the induction period include evaluation of the effect of CCX282-B on histopathological features of the ileal and colonic mucosa (using a histopathology score [D’Haens et al, 1998]), endoscopic appearance of the GI mucosa (using the Crohn’s Disease Endoscopic Index of Severity (CDEIS), on health-related quality of life (using the Short Form-36 version 2 [SF-36v2]), on serum C-reactive protein (CRP) concentrations, and determination of the preferred dose regimen of CCX282-B for treatment of subjects with moderate to severe Crohn’s disease.  The primary efficacy objective of the maintenance period (last 36 weeks) of this study is to determine the effect of CCX282-B compared to placebo on maintenance of response as measured by CDAI over a 36-week period. Secondary efficacy objectives of the maintenance period include evaluation of the effect of CCX282-B on health-related quality of life, on serum CRP concentrations, and on glucocorticoid use.  The primary safety objective of this study is to evaluate the safety and tolerability of CCX282-B administered orally for up to fifty two consecutive weeks for the treatment of subjects with moderate to severe Crohn’s disease. In addition, plasma samples will be collected to evaluate the population pharmacokinetic profile of CCX282-B.  Exploratory objectives include assessment of the effect of CCX282-B on biomarkers, e.g., cytokine expression in intestinal biopsies, and to evaluate the CCR9 receptor status. | | |
| **Methodology:** The study will consist of an induction period (first 12 weeks), an active treatment period (4 weeks), a maintenance period (36 weeks), and a follow-up period (4 weeks). This double-blind, placebo-controlled, parallel group study will enroll approximately 423 subjects with active, moderate to severe Crohn’s disease (CDAI at study entry between 250 and 450, inclusive), to obtain approximately 400 evaluable subjects for the induction period who either complete the study procedures through at least Study Day 29 or terminate from the study early due to a need for rescue medication. For the induction period, subjects will be assigned randomly in a 1.5:1:1:1 ratio to receive either placebo or one of three dose regimens of CCX282-B (250 mg or 500 mg daily, or 250 mg twice per day) administered for up to twelve consecutive weeks.  For the induction period of the study, subjects will visit the study center for screening procedures within 28 days prior to study entry. Subjects will record their Crohn’s disease symptoms daily for at least seven consecutive days during the Screening Period prior to their Randomization and from Study Day 1 daily throughout Study Day 84. Subjects will visit the study center for randomization and treatment initiation on Study Day 1, and for assessments on Study Days 15, 29, 57, and 85. Beginning on Study Day 1, subjects will self-administer an oral dose of blinded study medication twice daily for up to twelve consecutive weeks (i.e., through Study Day 84).  For the induction period of the study, CDAI scores will be determined during the Screening Period (prior to randomization) and on Study Days 29, 57, and 85. Serum CRP concentrations will be determined, with the subjects fasting for at least 8 hours, during the Screening Period, and on Study Days 1 (prior to administration of the first dose of study medication), 29, 57, and 85. Study subjects will complete the SF-36v2 on Study Day 1 (prior to administration of the first dose of study medication) and on Study Day 85. For subjects who are willing to undergo endoscopies, 2 endoscopic examinations of the colon and terminal ileum with biopsies will be performed. The first endoscopic examination will be done within 4 days after randomization and approximately 2 days prior to Study Day 1 (when the subject will receive the first dose of study drug). The second endoscopic examination will be done within approximately 3 days after Study Day 85 or within 3 days after early termination from the study. Safety evaluations will be performed at each visit to the study center. Plasma samples for pharmacokinetic analysis will be collected on Study Days 1 (at 2 time points following dosing), 29, 57, and 85, as well as at the time of any early termination. After completion of the induction period of the study, all subjects, irrespective of the study drug assignment for the induction period, will be eligible for a 4-week active treatment period during which they will all receive 250 mg CCX282-B twice daily (bid). Subjects who are in the 28-day follow-up period at the time of implementation of Amendment 1 of the protocol, will be allowed to enter the 4-week active treatment period following their Day 113F visit. These subjects may come in early for their Day 113F visit if they want to continue in the active treatment period of the study. Subjects who have completed the study by the time of implementation of Amendment 1 of the protocol, will also be allowed to enter the 4-week active treatment period if they are within 3 months after completing the study. These subjects must come to the study center for a Day 113F2 visit. At the end of the 4-week active treatment period, all subjects who are CDAI delta 70 responders (decrease from baseline, assessed during the Screening Period, of at least 70 points) will be randomized 1.5:1 to receive 250 mg CCX282-B bid or placebo in a blinded manner for a further 36-week maintenance period. Subjects who are CDAI delta 70 non-responders at the end of the 4-week active treatment period will not be eligible to continue in the 36-week maintenance period of the study. The CDAI delta 70 non-responders will be asked to return to the study center 4 weeks later for a follow-up visit. The procedures specified for Study Day 393 will be completed at that time. Subjects will be followed for safety for 28 days after receiving their final dose of study medication and will be terminated from the study at the completion of the Termination Visit conducted on Study Day 113F (for subjects who do not continue in the active treatment and maintenance periods), Study Day 393 (for subjects who have completed the maintenance period), or 28 days after the final dose of study medication in the case of an early termination. Whenever possible, any adverse events that are ongoing at discharge will be followed to resolution or stabilization. If a subject is taking glucocorticoids over the course of the study, the glucocorticoid dose could be tapered starting at Day 57 if the subject’s condition justifies a dose reduction. Tapering to a dose of zero must occur over a period not to exceed 6 weeks.  For the maintenance period of the study, subjects will visit the study center for assessments on Study Days 113 (after the 4-week CCX282-B treatment period), 141, 197, 253, 309, 365, and 393. Beginning on Study Day 85, subjects will self-administer an oral dose of 250 mg CCX282-B twice daily for 4 weeks. After the 4-week CCX282-B treatment period, beginning on Study Day 113, eligible subjects continuing in the maintenance period will self-administer an oral dose of blinded study medication twice daily for up to 36 consecutive weeks (i.e., through Study Day 364).  Starting at the Day 113 visit, an attempt must be made to taper the corticosteroid dose in all subjects who continue in the study and remain on corticosteroid therapy. This tapering to a dose of zero must occur over a period not to exceed 6 weeks. These corticosteroid dose reductions must be recorded in the electronic case report form (eCRF).  For the maintenance period of the study, CDAI scores and CRP concentrations will be determined on Study Days 113, 141, 197, 253, 309, and 365. Study subjects will complete the SF-36v2 on Study Days 113 and 365. | | |
| **Number of Subjects:** Up to approximately 423 subjects with moderate to severe Crohn’s disease will be entered into the study in order to obtain approximately 400 evaluable subjects for the induction period who either complete the study procedures through at least Study Day 29 or terminate from the study early due to a need for rescue medication. The sample size of approximately 133 evaluable subjects in the placebo group and 89 evaluable subjects per CCX282-B treatment group is based on approximately 25% of subjects in the placebo group achieving a clinical response (CDAI score decrease of at least 70) compared with 50% in at least 1 of the CCX282-B groups (overall alpha 0.05, power 90%, Holm’s step-down procedure for multiple comparisons).  It is estimated that, of the subjects who received CCX282-B during the 4-week active treatment period of the study and were CDAI delta 70 responders, at least 64 subjects in the placebo group and 96 subjects in the CCX282-B group in the maintenance period will be evaluable for the maintenance of response endpoints. | | |
| **Main Criteria for Inclusion:** Male or female subjects, at least 18 years old, who have active, moderate to severe Crohn’s disease with a history (radiographic or colonoscopic documentation) of small bowel and/or colonic disease. The CDAI recorded during the Screening Period must be between 250 and 450, inclusive. Fasting serum CRP concentration must be above 7.5 mg/L during the Screening Period. If a subject has been receiving therapy for Crohn’s disease (methotrexate, azathioprine, 6-mercaptopurine, and/or up to 20 mg per day of prednisone or prednisone-equivalent), s/he must have been on a stable treatment regimen for at least four weeks immediately prior to study entry and must be expected to continue this regimen throughout at least the first 196 days, except for glucocorticoids that must be tapered after the Day 113 visit. 5-ASA therapy is allowed during the study. If a female of childbearing potential, or if a male whose partner is a woman of childbearing potential, the subject must agree to use adequate contraception during the 364 days of administration of study medication. The subject must be willing and able to give written Informed Consent and comply with the requirements of the study protocol. No more than 100 cm of the subject’s small bowel should have been resected. If taking oral antibiotics chronically, the subject must have been using these continuously for at least 4 weeks prior to randomization and at stable doses for at least 2 weeks prior to randomization. | | |
| **Main Criteria for Exclusion:** If female, the subject is pregnant or breast feeding. The subject has a medical history of sensitivity to any of the components of the CCX282-B formulation (microcrystalline cellulose, polyvinyl pyrrolidone, sodium lauryl sulphate, colloidal silicon dioxide, crospovidone, or sodium stearyl fumarate). Use of cyclosporine, tacrolimus, sirolimus, or mycophenolate mofetil and/or greater than 20 mg prednisone or a prednisone-equivalent, parenteral glucocorticoids or corticotrophin, or any experimental treatment for Crohn’s disease during the four weeks prior to study entry. TNF inhibitor or natalizumab use during 12 weeks prior to study entry; patients with ileostomies, colostomies, or rectal pouches; history or presence of illicit drug use and/or alcohol abuse within the year prior to study entry; history or presence of any medical or psychiatric condition or disease, or laboratory abnormality that, in the opinion of the Investigator, may place the subject at unacceptable risk for study participation and completion; bowel surgery (other than appendectomy) within 12 weeks prior to randomization and/or planned or likely to require bowel surgery during the study; presence of symptomatic obstructive stricture; active tuberculosis, hepatitis B, C and/or human immunodeficiency virus (HIV) infection; history of any form of cancer within five years prior to study entry, with the exception of basal cell or squamous cell skin cancer, cervical carcinoma in situ, or breast carcinoma in situ that has been excised or resected completely and is without evidence of recurrence or metastasis. The subject has evidence of short bowel syndrome requiring enteral or parenteral nutritional supplementation or total parenteral nutrition. The subject has a history of infection requiring intravenous antibiotics, a serious local infection (eg, cellulitis or abscess), systemic infection (eg, pneumonia, septicemia), or gastrointestinal infection within 12 weeks of randomization. The subject has been diagnosed with ulcerative or indeterminate colitis. | | |
| **Test and Reference Products, Doses and Mode of Administration:** Study medication is supplied in kits (boxes) containing 3 bottles. Each bottle contains 30 hard gelatin capsules filled with either placebo or 250 mg CCX282-B. Each kit contains sufficient study medication for 30 days. For the induction period of the study, subjects will receive one kit on Study Days 1, 29, and 57. For the 4-week active treatment period, all subjects will receive one kit to provide 250 mg CCX282-B bid. For the maintenance period of the study, subjects will receive one kit of blinded study medication on Study Day 113 and two kits on Study Days 141, 197, 253, and 309. The placebo and CCX282-B capsules are identical in appearance. All subjects will be instructed to take one capsule from each of the first two bottles in the kit every morning and one capsule from the third bottle every evening, approximately 12 hours after the morning dose.   - Subjects randomized to the placebo group will take two placebo capsules every morning and one placebo capsule every evening approximately 12 hours after the morning dose; - Subjects randomized to the 250 mg CCX282-B daily group will take one 250 mg CCX282-B capsule and one placebo capsule every morning, and one placebo capsule in the evening, approximately 12 hours after the morning dose; - Subjects randomized to the 500 mg CCX282-B daily group will take two 250 mg CCX282-B capsules every morning, and one placebo capsule in the evening, approximately 12 hours after the morning dose; and - Subjects randomized to the 250 mg CCX282-B twice daily group will take one 250 mg CCX282-B capsule and one placebo capsule every morning, and one 250 mg CCX282-B capsule in the evening, approximately 12 hours after the morning dose.   The first dose of study medication will be administered while the subject is at the study center. Study drug will be taken with approximately 8 oz (240 mL) of water. There are no restrictions on dosing of study medication with regard to food consumption. | | |
| **Duration of Treatment and Observation:** Subjects will be screened for study eligibility within 28 days prior to study entry. Subjects will self-administer an oral dose of study medication twice daily for up to fifty two consecutive weeks (i.e., through Study Day 364). Subjects will be followed for safety for 28 days after receiving their final dose of study medication and will be terminated from the study at the completion of the Study Day 393 visit. Whenever possible, any adverse events that are ongoing at discharge will be followed-up to resolution or stabilization. | | |
| **Efficacy Assessments:** **For the induction period of the study**, subjects will record their Crohn’s disease symptoms for the previous 24 hours by calling an interactive voice response system (IVRS) every night before going to bed for at least seven consecutive days prior to randomization and daily from Study Day 1 throughout Study Day 84. CDAI scores will be determined during the Screening Period (prior to randomization), on Study Days 29, 57, and 85. CDEIS and histopathology scores will be determined prior to Study Day 1 and after Study Day 85 in subjects undergoing endoscopy. Serum CRP concentrations will be determined after an overnight fast of at least 8 hours during the Screening Period and on Study Days 1 (prior to administration of the first dose of study medication), 29, 57, and 85. SF-36v2 will be collected on Study Day 1 (prior to administration of the first dose of study medication) and on Study Day 85. For non-English-speaking subjects, only validated translations of the SF-36v2 instrument will be used in the study.  For the 4-week active treatment period, subjects will record their Crohn’s disease symptoms by calling the IVRS every night before going to bed. For the 36-week maintenance period of the study, subjects will record their Crohn’s disease symptoms by calling the IVRS every night before going to bed starting 10 days prior to each study visit. Serum CRP concentrations will be determined after an overnight fast of at least 8 hours on Study Days 113, 141, 197, 253, 309, 365, and 393. SF-36v2 will be collected on Study Days 113 and 365 or at early termination.  For subjects who do not continue in the active treatment period and maintenance period of the study, a Day 113F visit will be completed. At this visit, CDAI scores will be determined, CRP concentration will be assessed, and the SF-36v2 questionnaire will be completed. | | |
| **Efficacy Endpoints:** For the induction period of the study, the primary efficacy endpoint is the attainment of a clinical response (defined as a decrease from baseline in CDAI score of at least 70) at Day 57. An important secondary endpoint (conditional upon successful outcome of the primary endpoint) for the induction period is the attainment of clinical remission (defined as a CDAI score of ≤150) at Day 57. Other secondary efficacy endpoints for the induction period include: (1) the attainment of a clinical response (defined as a decrease from baseline in CDAI score of at least 70) at Days 29, 85, and 113F; (2) the attainment of clinical remission at Days 29, 85, and 113F; (3) the attainment of a clinical response (defined as a decrease from baseline in CDAI score of at least 100) at Days 29, 57, 85, and 113F; (4) the attainment of improvement in ileal histopathology score at the end of treatment; (5) the attainment of quiescence (score <4) based on ileal histopathology score at the end of treatment; (6) the attainment of improvement in colonic histopathology score at the end of treatment; (7) the attainment of quiescence (score <4) based on colonic histopathology score at the end of treatment; (8) change from baseline in ileal and colonic histopathology scores to the end of treatment; (9) the attainment of endoscopic improvement based on CDEIS by the end of treatment; (10) change from baseline in CDEIS to the end of treatment; (11) area under the curve of change from baseline in CDAI scores over time; (12) change from baseline to Days 29, 57, 85, and 113F, in CDAI score (and its subcomponents); (13) change from baseline to Day 85 and 113F in SF-36v2, overall score, as well as its subcomponents; (14) change from baseline to Days 29, 57, 85, and 113F in CRP; (15) the attainment of a clinical response (decrease from baseline in CDAI score of at least 70) plus a decrease from baseline in CRP at Days 29, 57, 85, and 113F; (16) the attainment of a clinical response (decrease from baseline in CDAI score of at least 70) plus a decrease from baseline in CDEIS at Day 85; (17) percentage of subjects on corticosteroids at baseline for whom the corticosteroid dose was tapered and sustained below baseline level through Day 85; (18) percentage of subjects requiring rescue medication due to inadequate treatment response. Exploratory endpoints include change from baseline in expression of inflammatory cytokine levels in the intestinal biopsy samples, and the CCR9 receptor status.  For the maintenance period of the study, the primary endpoint is the attainment of a CDAI delta 70 response at the end of the 4-week active treatment period of the study (from Day 1 to Day 113) AND maintenance of the CDAI response at Study Day 365. Loss of CDAI response during the maintenance period is defined as a CDAI increase of more than 70 points from the Day 113 value AND an absolute CDAI above 250 during any of the ten-day observation periods before each study visit from Day 141 to Day 365 or the need for intervention after Day 113. Secondary endpoints include: (1) Attainment of a CDAI delta 70 response at Day 113 and maintenance of the CDAI response at Days 141, 197, 253, 309, and 393; (2) Attainment of a CDAI delta 100 response at Day 113 and maintenance of the CDAI response at Days 141, 197, 253, 309, 365, and 393; (3) Attainment of remission at Day 113 (CDAI ≤ 150) and maintenance of sustained remission (CDAI ≤ 150) at Days 141, 197, 253, 309, 365, and 393; (4) Time to loss of response (in subjects who had a CDAI delta 70 response at Day 113); (5) Time to loss of response (in subjects who had a CDAI delta 100 response at Day 113); (6) Time to loss of remission (in subjects who had a CDAI ≤ 150 at Day 113); (7) change from Day 113 to Days 141, 197, 253, 309, 365, and 393 in CDAI; (8) Percentage of subjects on corticosteroids at Day 113 for whom the corticosteroid dose was tapered and sustained below Day 113 level through Day 365; (9) Percentage of subjects on corticosteroids at Day 113 for whom the corticosteroid therapy was subsequently stopped through Day 365; (10) change from baseline in CRP; (11) change from Day 113 in CRP; (12) change from baseline to Day 365 in SF-36v2, overall score, as well as its subcomponents; (13) change from Day 113 to Day 365 in SF-36v2, overall score, as well as its subcomponents; and (14) percentage of subjects requiring rescue medication due to inadequate treatment response. | | |
| **Pharmacokinetic Assessments:** Plasma samples for population pharmacokinetic analysis will be collected on Study Days 1 (at two time points), 29, 57, 85, 113, 141, 197, 252, 309, and 365, and at the time of any early termination. The time of a meal and time of study drug dosing prior to PK sample collection, as well as time of PK sample collection will be recorded on the days of PK sampling. | | |
| **Safety Assessments:** Safety assessments will be performed at each visit to the study center. Safety will be evaluated by periodic physical examinations, assessments of vital signs, clinical laboratory tests (including blood chemistry, hematology, and urinalysis), electrocardiograms (ECGs, including duration of QTc interval), and monitoring of adverse events. Subjects will be followed for safety for 28 days after receiving their final dose of study medication and will be terminated from the study at the completion of the Study Day 393 visit or 28 days after the final dose of study medication in the event of early termination. | | |
| **Statistical Methods:** For the induction period of the study, the primary efficacy hypothesis is that the CCX282-B dose groups do not differ in the true probability of achieving a clinical response (defined as a decrease in CDAI score of at least 70 points from baseline) or clinical remission (defined as a decrease in CDAI score to ≤150) on Study Day 57 compared with placebo treatment. Other efficacy hypotheses will be considered secondary. The primary efficacy analysis will be an intent-to-treat analysis; the primary efficacy analysis will be performed on all randomized subjects who have signed informed consent and received at least one dose of study medication. All statistical testing will be two-sided and, except for the primary efficacy endpoint, with an alpha level of 0.05. For the primary efficacy analysis, Holm’s step-down procedure for multiple comparisons will be used to control the familywise error rate (FWE) at 0.05.  The primary efficacy null hypothesis for the maintenance period is that the CCX282-B group (from the CDAI responder group following the induction period and active treatment period) does not differ in the true probability of achieving maintenance of response during the period from Day 113 to Day 365 compared to the placebo group (from the CDAI responder group following the induction period and active treatment period). Loss of CDAI response during the maintenance period is defined as a CDAI increase of more than 70 points from the Day 113 value, and an absolute CDAI value above 250 or the need for intervention at any time after Day 113. CDAI scores are collected in ten-day periods up to and including study visits on Days 141, 197, 253, 309, and 365. Maintenance period efficacy assessed at a particular visit requires that there be no loss of CDAI response in any of the preceding ten-day data collection periods. Other efficacy hypothesis will be considered secondary. The primary efficacy analysis will be an intent-to-treat analysis; the primary efficacy analysis will be performed on all subjects who have signed informed consent to participate in the maintenance period of the study, and received at least one dose of study medication during the maintenance period. An interim analysis may be conducted when all subjects participating in the maintenance period of the study have completed the Study Day 197 visit. All statistical testing will be two-sided. The alpha level for the interim analysis will be 0.01 and for the final analysis 0.04.  The primary analysis of categorical variables, eg, those based on a comparison of number of subjects in each response category between each CCX282-B group and the placebo group will be by stratified Mantel-Haenszel one-degree-of-freedom chi-square test, with the strata defined by geographic region. As secondary analyses, clinical response and clinical remission, if applicable, will also be evaluated using the Mantel-Haenszel test for stratified 2 x 2 tables, stratified by geographic region. Continuous variables, eg, those based on differences in change from baseline will be analyzed using an analysis of covariance ANCOVA with change from baseline as the dependent variable, baseline value as the covariate, and a factor for treatment. Area under the curve analysis of repeated measures observations of the CDAI score change from baseline will also be compared between CCX282-B groups and placebo using an analysis of variance (ANOVA).  The dose-response relationship will be evaluated by the area under the curve analyses, as well as visual inspection of the results from the primary and secondary endpoints. Dose selection will be based on both efficacy and safety results.  Tabulations and descriptive statistics will be provided by treatment group for all safety data. All subjects randomized to treatment and receiving at least one dose of study medication will be included in the safety analysis, according to the treatment actually received.  Population pharmacokinetic parameters will be calculated based on plasma CCX282 concentrations at the time of sample collection in relation to time of administration of the most recent dose of study medication as well as time of food consumption. Plasma levels of CCX562 and CCX459 (minor metabolites of CCX282) as well as other identified metabolites will also be determined and population PK parameters calculated. | | |

Time and Events Table (for Induction Period)

|  | **28-Day Screening Period** | | | | | | **Random-ization (Study Entry)**^8^ | **Endo-scopy** | **Day 1** | | | **Day 15** | **Day 29** | **Day 57** | **Day 85 or Early Termin-ation** | **Day 113F ^14^** | **Day 113F2 ^15^** |
| --- | --- | --- | --- | --- | --- | --- | --- | --- | --- | --- | --- | --- | --- | --- | --- | --- | --- |
| Informed consent | X |  |  |  |  |  |  |  |  |  |  |  |  |  |  | **X** | **X** |
| Demographics, Medical and Medication History |  | X |  |  |  |  |  |  |  |  |  |  |  |  |  |  |  |
| Concomitant medications |  | X |  |  |  |  |  |  | X^10^ |  |  | X | X | X | X | X | **X** |
| Daily recording of Crohn’s disease symptoms for 7 consecutive days |  |  | X |  |  |  |  |  |  |  |  |  |  |  |  |  |  |
| Complete physical examination including Weight, Height^1^ |  |  |  | X |  |  |  |  |  |  |  |  |  |  | X |  |  |
| Limited physical examination |  |  |  |  |  |  |  |  | X^11^ |  |  | X | X | X |  | X | **X** |
| Vital signs^2^ |  |  |  | X |  |  |  |  | X^10^ |  |  | X | X | X | X | X | **X** |
| Hematology |  |  |  | X |  |  |  |  | X^10,11^ |  |  | X | X | X | X | X | **X** |
| C-Reactive Protein^3^ |  |  |  | X |  |  |  |  | X^10^ |  |  |  | X | X | X | X | **X** |
| CDAI and CRP eligibility determination |  |  |  |  | X |  |  |  |  |  |  |  |  |  |  |  |  |
| ECG |  |  |  |  |  | X |  |  | X^10,12^ |  | X^12^ |  |  |  | X |  |  |
| Serum Chemistry |  |  |  |  |  | X |  |  | X^10,11^ |  |  | X | X | X | X | X | **X** |
| Urinalysis |  |  |  |  |  | X |  |  | X^10,11^ |  |  | X | X | X | X | X | **X** |
| HIV, HBV, HCV screen |  |  |  |  |  | X |  |  |  |  |  |  |  |  |  |  |  |
| TB screen^4^ |  |  |  |  |  | X |  |  |  |  |  |  |  |  |  |  |  |
| Chest radiographs if TB test is positive |  |  |  |  |  | X |  |  |  |  |  |  |  |  |  |  |  |
| Drugs of abuse screen |  |  |  |  |  | X |  |  |  |  |  |  |  |  |  |  |  |
| Pregnancy test^5^ |  |  |  |  |  | X |  |  | X^10,11^ |  |  |  |  |  |  |  |  |
| Randomization |  |  |  |  |  |  | X |  |  |  |  |  |  |  |  |  |  |
| Endoscopy and biopsies |  |  |  |  |  |  |  | X^9^ |  |  |  |  |  |  | X^13^ |  |  |
| Study Drug Dispensing |  |  |  |  |  |  |  |  |  | X |  |  | X | X |  | **X** | **X** |
| Daily Recording of Crohn’s disease symptoms^6^ |  |  |  |  |  |  |  |  |  |  | X | → | → | → | → | X |  |
| CDAI recording |  |  |  |  | X |  |  |  |  |  |  |  | X | X | X | X |  |
| Plasma sample collection for PK^7^ |  |  |  |  |  |  |  |  |  |  | X |  | X | X | X |  |  |
| SF-36v2 |  |  |  |  |  |  |  |  | X^10^ |  |  |  |  |  | X | X |  |
| Backup plasma and serum collection |  |  |  |  |  |  |  |  | X^10^ |  |  |  |  |  | X | X |  |
| Blood sample for CCR9 assessment |  |  |  |  |  |  |  |  | X |  |  |  |  |  |  |  |  |
| Adverse event recording |  |  |  |  |  |  |  |  |  |  | X | X | X | X | X | X | **X** |

^1^ Height measured at Screening only

^2^  Assessment of heart rate, body temperature, blood pressure (supine after a rest period of 3 minutes)

^3^ Fasting (at least 8 hours) serum sample required for C-reactive protein assay

^4^ Chest X rays could be done to rule out TB. Alternatively, a tuberculin skin test, QuantiFERON®-TB Gold test, or T-SPOT™.*TB* test could be done,

and if any of these tests are is positive, chest radiographs must be done to rule out active tuberculosis.

^5^ For women of childbearing potential: serum pregnancy test during Screening (assay by central laboratory). If more than 14 days have elapsed between screening

assessment and Day 1 visit, a local urine pregnancy test should be performed on Study Day 1.

^6^ Completed daily before going to bed and recorded via IVRS starting on Study Day 1 and continuing daily throughout Study Day 112

^7^ Blood samples will be taken at 2 time points after receiving the first dose on Day 1. These 2 samples will be taken at least 4 hours apart. Blood samples for the

other study visits could be taken any time before or after dosing on these study days. The time of meals and time of dosing prior to all PK sample collections,

as well as time of PK sample collections will be recorded on the days of PK sampling.

^8^  For subjects not undergoing endoscopy, the Randomization visit and Study Day 1 visit will occur on the same day

^9^  In subjects willing to undergo endoscopies, an endoscopic examination will be performed within 4 days after randomization and approximately 2 days before

receiving the first dose on Day 1; Endoscopy may be performed on the day of randomization, but only after the randomization procedure has been completed.

^10^ Procedures to be performed after randomization to treatment but prior to administration of first dose of study medication

^11^ Not necessary to repeat if screening assessment was done within 14 days prior to Study Day 1

^12^ ECG to be performed before dosing and approximately 3 to 4 hours after dosing on Day 1.

^13^ Endoscopy to be performed within 3 days after Day 85.

^14^ Day 113F follow-up visit applies to: (1) subjects who are in the follow-up period when Amendment 1 is implemented, and who want to continue in the active period of the study, and (2) subjects who complete Day 85 and do not want to continue in the study. Subjects who discontinue the study prior to Day 85 will be asked to return 28 days later and Study Day 393 follow-up procedures will be completed.

^15^ Day 113F2 follow-up visit only applies to subjects who have completed the original protocol and want to continue in the study.

Time and Events Table (for Active Treatment and Maintenance Periods)

|  | Day 85 | Day 113 | Day 141 | Day 197 | Day 253 | Day 309 | Day 365 or Early Term | Day 393 |
| --- | --- | --- | --- | --- | --- | --- | --- | --- |
| Informed consent ^1^ | X |  |  |  |  |  |  |  |
| CDAI determination for maintenance period eligibility assessment |  | X |  |  |  |  |  |  |
| Concomitant medications | X | X | X | X | X | X | X | X |
| CDAI recording ^2^ | X | X | X | X | X | X | X | X |
| Complete physical exam including weight | X | X |  | X |  |  | X |  |
| Limited physical examination including weight |  |  | X |  | X | X |  | X |
| Vital signs ^3^ | X | X | X | X | X | X | X | X |
| Hematology | X | X | X | X | X | X | X | X |
| C-Reactive Protein ^4^ | X | X | X | X | X | X | X | X |
| Serum Chemistry | X | X | X | X | X | X | X | X |
| Urinalysis | X | X | X | X | X | X | X | X |
| Study Drug Dispensing ^5^ | X | X | X | X | X | X |  |  |
| SF-36v2 | X | X |  |  |  |  | X |  |
| Plasma sample collection for PK | X | X | X | X | X | X | X |  |
| Backup plasma and serum collection | X | X | X | X |  |  | X |  |
| Adverse event recording | X | X | X | X | X | X | X | X |

^1^ Obtain signed informed consent prior to continuing in active and maintenance periods of the study, if the subject has previously only signed consent for

the induction period of the study. If a subject does not want to continue in the study, Day 113F procedures must be completed 28 days after taking the

last dose of study medication.

^2^ Completed daily before going to bed and recorded via IVRS from Day 85 to Day 113. Completed daily before going to bed and recorded via IVRS

starting 10 days prior to Study Days 141, 197, 253, 309, 365, and 393.

^3^  Assessment of heart rate, body temperature, blood pressure (supine after a rest period of 3 minutes)

^4^ Fasting (at least 8 hours) serum sample required for C-reactive protein assay

^5^ One kit for treatment with 250 mg CCX282-B twice daily will be dispensed to all subjects on Study Day 85 (or 113F or 113F2 for subjects who want

to continue the study); one kit of blinded study medication (either 250 mg CCX282-B twice daily or placebo) will be dispensed on Study Day 113; two kits of

blinded study medication (either 250 mg CCX282-B twice daily or placebo) will be dispensed on Study Days 141, 197, 253, and 309.

**Study Schema**

Induction

Period

Active

Treatment

Period

Maintenance

Period

Day 1- 85

Day 85 - 113

Day 113 - 365

Follow-Up

Period

Day 365 - 393

Randomization 1.5:1:1:1

Placebo

250 mg/d CCX282-B

500 mg/d CCX282-B

250 mg bid CCX282-B

250 mg bid

CCX282-B

Randomization 1:1.5

Placebo

250 mg bid CCX282-B

CDAI delta 70

Responders

Discontinue

CDAI delta 70

Non-Responders

No Study Drug
