# Supplementary material for: A Randomized Controlled Trial of the Efficacy and Safety of CCX282-B, an Orally-Administered Blocker of Chemokine Receptor CCR9, for Patients with Crohn’s Disease
Source: PLoS One. 2013 Mar 20;8(3):e60094. doi: 10.1371/journal.pone.0060094 (PMC3603920; doi:10.1371/journal.pone.0060094)
Supplement: Appendix S1 — Study Centers and Ethics Committees. (DOC) [file pone.0060094.s005.doc]

**Appendix S1**

Investigators

Apart from the primary authors, the following investigators form the Prospective Randomized Oral-Therapy Evaluation in Crohn’s disease Trial-1 (PROTECT-1) Study Group: **Australia**— D. Badov, Bayside Gastroenterology, Frankston; W. Connell, St. Vincent’s Hospital Melbourne, Fitzroy; S. Edwards, Flinders Medical Center, Bedford Park; P. Gibson, Box Hill Hospital, Box Hill; R. Leong, Bankstown-Lidcombe Hospital, Bankstown; F. Macrae, Royal Melbourne Hospital, Parkville; B. Mitchell, Launceston General Hospital, Launceston; G. Radford-Smith, Royal Brisbane and Women’s Hospital, Herston; **Austria**— A. Kaser, Universitätsklinik für Innere Medizin, Innsbruck; Belgium— M. De Vos, UZ Gent, Gent; **Brazil**— H. Amarante, Hospital de Clínicas da UFPR, Curitiba; J. Fernandes, Irmandade Santa Casa de Misericórdia de Santos, Santos; W. Ferreira, Centro Petropolitano de Reumatologia e Fisioterapia, Centro Petrópolis; C. Francisconi, Hospital de Clínicas de Porto Alegre, Porto Alegre; M. Machado, Hospital São Lucas – PUCRS, Porto Alegre; E. Rolim, Irmandade Santa Casa de Misericórdia de São Paulo, São Paulo; A. Sipahi, Hospital das Clínicas da Faculdade de Medicina da USP, São Paulo; F. Steinwurz, Hospital Israelita Albert Einstein, São Paulo; C. Uzum, Santo André Diagnósticos e tratamento, Santo André; A. Ximenes, Hospital Geral de Goiânia, Goiânia; C. Zaltman, Setor de Gastroenterologia, Cidade Universitária, Rio de Janiero; **Bulgaria**— R. Draganova, 4 MHAT, Sofia; K. Katzarov, Military Medical Academy, Sofia; N. Kostov, V City Hospital, Sofia; I. Kotzev, UMHAT “St. Marina”, Varna; Z. Krastev, UMHAT “St. Ivan Rilsky”, Sofia; G. Krasteva, MHAT, Ruse; S. Stoynov, UMHAT Queen Joanna, Sofia; K. Tchernev, UMHAT “Alexandrovska”, Sofia; **Canada**— F. Anderson, Lair Center, Vancouver; A. Cohen, Jewish General Hospital, Montreal; R. Panaccione, Health Science Center, Calgary; H. Steinhart, Mt. Sinai Hospital, Toronto; **Czech Republic**— V. Compel, Private Gastroenterology Center, České Budejovice; L. Douda, Regional Hospital Tabor, Tabor; V. Hajas, Private endoscopy practice, Benesov u Prahy; M. Lukas, ISCARE IVF a.s., Prague; V. Prochazka, Il. Interni Klinika Endoskopie, Olomouc; D. Pulgretova, Regional Hospital Pribram, Pribram; J. Spicak, IKEM Klinika hepatogastroenterologie, Praha; Z. Zadorova, II. Interni klinika Fakultní nemocnice Královské, Praha; **Denmark**—J. Dahlerup, Århus University Hospital, Århus; J. Fallingborg, Ålborg Hospital South, Ålborg; A. Mertz-Nielsen, Hvidovre Hospital, Hvidovre; **France**—J-F Colombel, Service d'Hépato-Gastroentérologie CHRU - Hôpital Huriez, Lille; X. Hebuterne, Hôpital l'Archet 2, Nice; **Germany**— Y. Dörffel, Universitätsmedizin Berlin, Berlin; U. Seidler, Medizinische Hochschule Hannover Abteilung Gastroenterologie, Hannover; **Hungary**— M. Varga, Réthy Pál Kórház III. sz. Belgyógyászati Osztály, Békéscsaba; M. Zeher, Debreceni Egyetem OEC III. sz. Belgyógyászati Klinika, Debrecen; **Israel**— I. Dotan, The Tel-Aviv Sourasky Medical Center, Tel Aviv; A. Fich, Soroka Medical Center, Beer-Sheva; E. Goldin, Hadassah Ein-Karem Medical Center, Jerusalem; F. Konikoff, Meir Hospital, Kfar-Saba; A. Lavy, Bnai Zion MC, Haifa; E. Melzer, Kaplan Medical Center, Rechovot; E. Scapa, Assaf Harofeh Medical Center, Trzifin; **The Netherlands**— R. Ouwendijk, Ikazia Ziekenhuis Maag-, Darm-, en Leverziekten, Rotterdam; M. Otten, Ringweg Randenbroek, Amersfoort; J. Vecht, Isala Klinieken Locatie Sophia, Zwolle; **Poland**—K. Celinski, Samodzielny Publiczny Szpital Kliniczny Nr 4 Katedra I Klinika Gastroenterologii, Lublin; J. Gil, Wojskowy Insytutu Medyczny Klinika Gastroenterologii Centralnego Szpitala Klinicznego MON, Warszawa; Z. Hebzda, 5 Wojskowy Szpital Kliniczny z Poliklinika Klinika Chorob Wewnetrznych I Gastroenterologii, Krakow; M. Horynski, SOPMED Niepubliczny Zakład Opieki Zdrowotnej, Sopot; L. Jackowski, NZOZ-GCP Dobra Praktyka Lekarska, Grudziądz; M. Klopocka, SPZOZ Wojewodzki Szpital. im. Dr J. Biziela Poradnia Gastroenterologiczna, Bydgoszczy; K. Marlicz, Klinika Gastroenterologii i Chorób Wewnętrznych, Samodzielny Publiczny Szpital Kliniczny nr 1 im. T. Sokołowskiego Pomorskiej Akademii Medycznej, Szczecin; L. Paradowski, Klinika Gastroenterologii i Hepatologii, SPZOZ Akademicki Szpital Kliniczny im. J. Mikulicza-Radeckiego we Wroclawiu, Wrocaw; J. Rudzinski, 10 Wojskowy Szpital Kliniczny z Poliklinika Oddzial Kliniczny Gasteroenterologii, Bydgoszcz; G. Rydzewska, CSK MSWiA Klinika Gastroenterologii, Warszawa; **South Africa**— N. Aboo, Parklands Medical Center, Durban; L. Fouché, Private Practice, Johannesburg; F. Kruger, Louis Leipoldt Medical Center, Cape Town; S. Schmidt, Quatro Clinical Trial Institute, Cape Town; P. van Eeden, Private Practice, Panorama Medi-Clinic, Cape Town; G. Watermeyer, Groote Schuur Hospital, Cape Town; **Sweden**—E. Hertevig, Endokrinologi/Gastroenterologi Universitetssjukhuset, Lund.

Ethics Committees

The following ethics committees reviewed and approved the clinical trial: **Australia**— D. Badov: Peninsula Health Human Ethics Committee; W. Connell: St. Vincent’s Hospital (Melbourne) Ltd. HREC D; S. Edwards: Flinders Clinical Research Ethics Committee; P. Gibson: Eastern Health Research and Ethics Committee; D. Hetzel: Royal Adelaide Hospital Research Ethics Committee; R. Leong: The Sydney South West Area Health Service Human; F. Macrae: Melbourne Health Research Directorate; B. Mitchell: Human Research Ethics Committee (Tasmania) Network; G. Radford-Smith: Royal Brisbane and Women’s Hospital Human Research Ethics Committee; **Austria**— A. Kaser, W. Reinisch: Ethik-Kommission Medizinische Universität Wien; **Belgium**— M. De Vos: Ethisch Comité; S. Vermeire: Commissie Medische Ethiek; **Brazil**— H. Amarante, M. Baffuto, J. Fernandes, W. Ferreira, C. Francisconi, M. Machado, E. Rolim, A. Sipahi, F. Steinwurz, C. Uzum, A. Ximenes, C. Zaltman: Ministério da Saúde Comissão Nacional de Ética em Pesquisa - CONEP; **Bulgaria**— R. Draganova: 4 MHAT; K. Katzarov: Military Medical Academy; N. Kostov: V City Hospital; I. Kotzev: UMHAT “St. Marina” Varna; Z. Krastev: UMHAT “St. Ivan Rilsky”; G. Krasteva: MHAT-Ruse; S. Stoynov: UMHAT Queen Joanna; K. Tchernev: UMHAT “Alexandrovska”; **Canada**— F. Anderson: IRB Services; A. Cohen: Jewish General Hospital; R. Panaccione: Conjoint Medical Bioethics Committee, University of Calgary; H. Steinhart: Mt. Sinai Hospital Research Ethics Board; **Czech Republic**— V. Compel: Local Ethics Committee of the Hospital Ceske Budejovice; L. Douda: Local Ethics Committee of the Regional Hospital Tabor; V. Hajas: Ethics Committe of the Faculty Hospital Olomouc; M. Lukas: Local Ethics Committee ISCARE IVF a.s.; V. Prochazka: Ethics Committee of the Faculty Hospital Olomouc; D. Pulgretova: Local EC of Regional Hospital Pribram; J. Spicak: Ethics Committee of the IKEM and FTNsP; T. Vaňásek: Ethics Committee of the Faculty Hospital in Hradec Kralove; Z. Zadorova: Ethics Committee of the Faculty Hospital Kralovske; **Denmark**— J. Dahlerup, J. Fallingborg, A. Mertz-Nielsen, O. Nielsen: Den Videnskabsetiske Komité for Region; **France**— J-F Colombel, X. Hebuterne: Comité de Protection des Personnes; **Germany**— Y. Dörffel, S. Howaldt, S. Schreiber, U. Seidler: Ethik-Kommission der Medizinischen; **Hungary**— I. Rácz: Ethkai Bizottsága; M. Varga: Réthy Pál Kórhaz-Rendelöintézet, Intézeti Kutatásetikai Bizottság; M. Zeher: Debreceni Egyetem OEC Kutatásetikai Bizottsaga; **Israel**— I. Dotan: Tel Aviv Sourasky Medical Center Ethics Committee; A. Fich: Soroka Medical Center Ethics Committee; E. Goldin: Hadassah Ein Karem Medical Center Ethics Committee; F. Konikoff: Meir Medical Center Ethics Committee; A. Lavy: Bnai Zion Ethics Committee; E. Melzer: Kaplan Medical Center Ethics Committee; Y. Niv: Rabin Medical Center Ethics Committee ; E. Scapa: Assaf Harofeh Medical Center Ethics Committee**; The Netherlands**— M. Otten, R. Ouwendijk, J. Vecht: Toetsingscommissie Wetenschappelijk; **Poland**— K. Celinski, J. Gil, Z. Hebzda, M. Horynski, L. Jackowski, M. Klopocka, K. Marlicz, L. Paradowski, R. Petryka, J. Rudzinski, G. Rydzewska: Komisja Bioetyczna Pomorskiej Akademii Medycznej w Szczecinie; **South Africa**— N. Aboo, L. Fouché, F. Kruger, S. Schmidt, P. van Eeden: South African Medical Association; G. Watermeyer: UCT Research Ethics Committee; **Sweden**— E. Hertevig, P. Karlén: Regionala Etikprövningsnämnden i Lund; **United Kingdom**— S. Keshav: West Glasgow Ethics Committee 1.
